# Fast Dissemination of New HIV-1 CRF02/A1 Recombinants in Pakistan

**DOI:** 10.1371/journal.pone.0167839

**Published:** 2016-12-14

**Authors:** Yue Chen, Bhavna Hora, Todd DeMarco, Sharaf Ali Shah, Manzoor Ahmed, Ana M. Sanchez, Chang Su, Meredith Carter, Mars Stone, Rumina Hasan, Zahra Hasan, Michael P. Busch, Thomas N. Denny, Feng Gao

**Affiliations:** 1 Duke Human Vaccine Institute, Department of Medicine, Duke University Medical Center, Durham, North Carolina, United States of America; 2 Bridge Consultants Foundation, Karachi, Pakistan; 3 Blood Systems Research Institute, San Francisco, California, United States of America; 4 Department of Pathology, Aga Khan University, Karachi, Pakistan; 5 Department of Microbiology, Aga Khan University, Karachi, Pakistan; University of Malaya, MALAYSIA

## Abstract

A number of HIV-1 subtypes are identified in Pakistan by characterization of partial viral gene sequences. Little is known whether new recombinants are generated and how they disseminate since whole genome sequences for these viruses have not been characterized. Near full-length genome (NFLG) sequences were obtained by amplifying two overlapping half genomes or next generation sequencing from 34 HIV-1-infected individuals in Pakistan. Phylogenetic tree analysis showed that the newly characterized sequences were 16 subtype As, one subtype C, and 17 A/G recombinants. Further analysis showed that all 16 subtype A1 sequences (47%), together with the vast majority of sequences from Pakistan from other studies, formed a tight subcluster (A1a) within the subtype A1 clade, suggesting that they were derived from a single introduction. More in-depth analysis of 17 A/G NFLG sequences showed that five shared similar recombination breakpoints as in CRF02 (15%) but were phylogenetically distinct from the prototype CRF02 by forming a tight subcluster (CRF02a) while 12 (38%) were new recombinants between CRF02a and A1a or a divergent A1b viruses. Unique recombination patterns among the majority of the newly characterized recombinants indicated ongoing recombination. Interestingly, recombination breakpoints in these CRF02/A1 recombinants were similar to those in prototype CRF02 viruses, indicating that recombination at these sites more likely generate variable recombinant viruses. The dominance and fast dissemination of new CRF02a/A1 recombinants over prototype CRF02 suggest that these recombinant have more adapted and may become major epidemic strains in Pakistan.

## Introduction

Since the first case of AIDS in Pakistan was reported in 1987 [1], the estimated number of people infected with HIV has increased to ~87,000 in 2012 [2, 3]. Like other Asia countries, Pakistan experiences a comparable HIV epidemic trend from “low prevalence, high risk” to “concentrated” epidemic in the early to mid-2000s [4]. Although Pakistan currently has a low HIV prevalence (<0.1%) in generation population [2], a widespread of HIV epidemic is predicted, primarily due to high-risk practices among three populations; people who inject drug (PWID), Hijra (Transgender) sex workers (HSW), and men who have sex with men (MSM) [4, 5]. Importantly, only 50% of PWIDs are tested for HIV-1 infection [6], while more than half of HSWs (57.6%) never used condoms [7, 8]. These high-risk factors have accelerated HIV-1 epidemic in Pakistan.

A number of subtypes and circulating recombinant forms (CRFs) have been reported in Pakistan [9–11], but no nationwide surveys were performed to systematically study distribution of HIV-1 subtypes and recombinants in the country. Examination of sequences available from the Los Alamos HIV Sequence Database (www.hiv.lanl.gov) showed that subtype A1 was most reported (84.3%), while subtype B, CRF02, A1/G recombinant and others accounted for 8.7%, 2.0%, 2.6% and 2.4%, respectively. However, all previous molecular epidemic surveys were carried out with small fragments of the *gag*, *pol* or *env* gene. No full length HIV-1 genome sequences have been obtained for viruses circulating in Pakistan. Thus, the distribution of subtypes or CRFs in Pakistan may not be accurately assessed by those partial gene sequences since the large portion of the viral genome are not analyzed. It is important to characterize HIV-1 whole genome sequences to better understand if new recombinants are generated and became more prevalent strains in Pakistan.

To fully understand what viruses are circulating in Pakistan, we analyzed near full length genome (NFLG) sequences from plasma samples from 34 HIV-1-infected individuals in Karachi, Pakistan. Phylogenetic and recombination analyses showed that new CRF02/A1 recombinants predominated the prototype CRF02 viruses while subtype A1 viruses still dominated the virus population in Pakistan. Our results indicate that new CRF02/A1 recombinants may become major strains and full length genome sequences are required to accurately monitor distribution of subtypes, CRFs, and URFs in Pakistan.

## Materials and Methods

### Generation of near full-length HIV-1 genome

All newly diagnosed HIV infected individuals who registered with Community Home Based Care (CHBC) of People Living With HIV/AIDS program between 2014 and 2015 were invited to participate in the study. Plasma samples were collected from 40 subjects who gave written informed consent. The study was approved by the ethics committee of Bridge Consultants Foundation and by the Duke University Institutional Review Board. Viral RNA was extracted from 400 μL of 28 plasma samples using EZ1 Virus Mini Kit v2.0 (Qiagen, Valencia, CA) and subject to cDNA synthesis using Superscript III Reverse Transcriptase (Invitrogen, Carlsbad, CA) with primer 1.R3.B3R (5’-ACTACTTGAAGCACTCAAGGCAAGCTTT ATTG -3’ HXB2 nt9611-9642) and 07Rev9 (5'-CTTCCTGCCATAGGAGATGCCTAA-3' nt 5957–5980) for 3'- and 5'-half HIV-1 genomes, respectively. The 3’-half and 5’-half genomes were obtained as bulk PCR products for each virus as previous described [12]. Six viruses (PK006, PK012, PK013, PK014, PK015 and PK030) were isolated from plasma by short-term co-culturing with peripheral blood mononuclear cells (PBMC) from HIV-1 negative donors [13]. Viral RNA was extracted form the cell culture supernatants and NFLGs were obtained by amplifying two overlapping half genomes for PK006, PK013 and PK015 or directly sequenced using TruSeq RNA and DNA Library Preparation Kit v2 (Illumina, San Diego, CA) for PK012, PK014 and PK030.

### Sequence analysis

PCR amplicons and TruSeq RNA libraries were quantified using qPCR with KAPA Library Quantification Kit Illumina platform (Kapa Biosystems, Wilmington, MA). The PCR amplicon or TruSeq library from each sample was barcoded and then sequenced on MiSeq (Illumina, San Diego, CA) using the MiSeq Reagent Nano kit v2 (300 bp). The average coverage was 500 and 8000 for each base for PCR amplicons and TruSeq libraries, respectively. The final consensus sequence from each library was obtained by assembling raw sequences reads using Geneious software (Biomatters, Auckland, New Zealand) or High-performance Integrated Virtual Environment (HIVE) [14].

The final sequences were aligned together with subtype reference sequences from HIV database in Los Alamos (www.hiv.lanl.gov) using CLUSTAL W [15] and manual adjustment for optimal alignment was done using SEAVIEW. Subtypes of newly characterized HIV-1 genomes were determined by phylogenetic tree analysis using the neighbor-joining (NJ) method with Kimura two-parameter model [16, 17], and the reliability of topologies was estimated by bootstrap analysis with 1000 replicates. Recombination patterns in newly characterized HIV-1 genomes were initially analyzed by the jumping profile Hidden Markov Model (jpHMM; http://jphmm.gobics.de/submission_hiv.html) [18]. The recombination breakpoints were confirmed by BootScan implemented in Simplot version 3.5.1 [19]. The recombination pattern map for each virus was generated using RecDraw [20].

### Molecular evolution clock analysis

Neighbor-joining phylogenetic tree was first analyzed with TempEst v1.5 (http://tree.bio.ed.ac.uk/software/tempest/) to determine the temporal signal for reliable estimation of MCRA before sequences were analyzed in BEAST [21]. The divergence times for subtype A1a and CRF02 were estimated using Bayesian Markov Chain Monte Carlo (MCMC) approach available in the BEAST v1.8.2 package. Both strict and relaxed (uncorrelated lognormal) molecular clocks were enforced under the GTR and HKY nucleotide substitution models [22], respectively, with a gamma-distribution model of among site rate heterogeneity (with four rate categories)[23]. Each MCMC analysis was run for 50 million steps and sampled every 10,000 states. Posterior probabilities were calculated with a 10% burn-in and checked for convergence using Tracer v1.6. The maximum clade credibility tree was generated using Tree Annotator v1.8.2 available in BEAST and FigTree 1.4.2 was used for visualization of the annotated trees [24].

### Nucleotide Sequence Accession numbers

The GenBank accession numbers for all sequences generated in this study are KX232594-KX232629.

## Results

### Characterization of plasma samples

HIV-1 infection Fiebig stages were determined based on the detection of viral genomes and HIV-1 specific antibodies in plasma as previously described [25]. One sample was at Fiebig stage V. Two could not be clearly separated between Fiebig stages V and VI (V/VI). Thirty were at Fiebig stage VI (Table 1). Limiting-Antigen Avidity (LAg) avidity was also performed to confirm if any subjects were recently infected. Thirty-one samples were found to be from long-term infections while PK009 and PK030 samples were from recent infections. The infection status could not be determined for PK032 since there was not enough plasma available for analysis. These results showed that 31 viruses were collected during chronic HIV-1 infection while two viruses were collected at early infection stage.

**Table 1:** Demographic characteristics of HIV-1 infected individuals.

Subject	Subtype	Viral Load (Copies/ml)	CD4 Count (cells/mm^3^)	Fiebig Stage	LAg Classification	Age	Marital Status	Transmission Route
PK001	A1	70,000	93	VI	LT	27	Single	Heterosexual
PK002	A1	488,000	271	VI	LT	43	Married	Heterosexual
PK003	CRF02/A1	13,600	478	VI	LT	30	Married	PWID
PK004	A1	464,000	426	VI	LT	20	Single	Heterosexual
PK006^#^	CRF02/A1	710,000	252	VI	LT	24	Single	PWID
PK007	A1	106,000	89	VI	LT	22	Married	PWID
PK008	CRF02/A1	625,000	397	VI	LT	27	Married	PWID
PK009	C	173,000	609	V/VI	Recent	22	Single	MSM
PK011	CRF02/A1	8,750	310	VI	LT	37	Married	PWID
PK012^#^^†^	CRF02/A1	84,000	336	VI	LT	34	Married	PWID
PK013^#^	A1	193,000	547	VI	LT	30	Married	PWID
PK014^#^^†^	A1	3,620	492	VI	LT	22	Married	PWID
PK015^#^	CRF02/A1	300,000	341	VI	LT	39	Widower	PWID
PK016	A1	13,000	297	VI	LT	20	Single	PWID
PK017	A1	119,000	347	VI	LT	26	Married	PWID
PK018	A1	88,500	282	VI	LT	30	Married	PWID
PK019	CRF02_AG	760,000	205	VI	LT	28	Single	PWID
PK020	CRF02/A1	208,000	583	VI	LT	33	Single	PWID
PK021	A1	93,500	98	VI	LT	48	Married	PWID
PK023	CRF02/A1	65,500	490	VI	LT	47	Married	PWID
PK024	CRF02_AG	6,050	823	VI	LT	30	Married	PWID
PK025	CRF02/A1	26,900	437	VI	LT	na	na	na
PK026	A1	5,350	104	VI	LT	22	Single	PWID
PK027	A1	111,500	na	VI	LT	30	Single	PWID
PK030^#^^†^	A1	99,000	498	VI	Recent	26	Married	PWID
PK031	A1	225,500	745	VI	LT	18	Single	PWID
PK032	CRF02_AG	6,400	na	na	na	18	Single	PWID
PK033	CRF02/A1	425,500	494	V	LT	25	Married	PWID
PK034	A1	4,665	1208	VI	LT	21	Single	PWID
PK035	CRF02_AG	461,500	339	VI	LT	32	Seperated	PWID
PK036	A1	96,500	299	V/VI	LT	30	Divorced	PWID
PK038	CRF02_AG	456,000	223	VI	LT	24	Single	PWID
PK039	CRF02_AG	60,000	346	VI	LT	26	Married	PWID
PK040	CRF02/A1	111,000	222	VI	LT	46	Married	PWID

# PBMC-derived viruses

† Near full-fength genome sequences were obtained by TruSeqTM RNA sequencing

na: data not available

### The majority of subtype A viruses in Pakistan were the result of a single introduction

**Fig 1:**
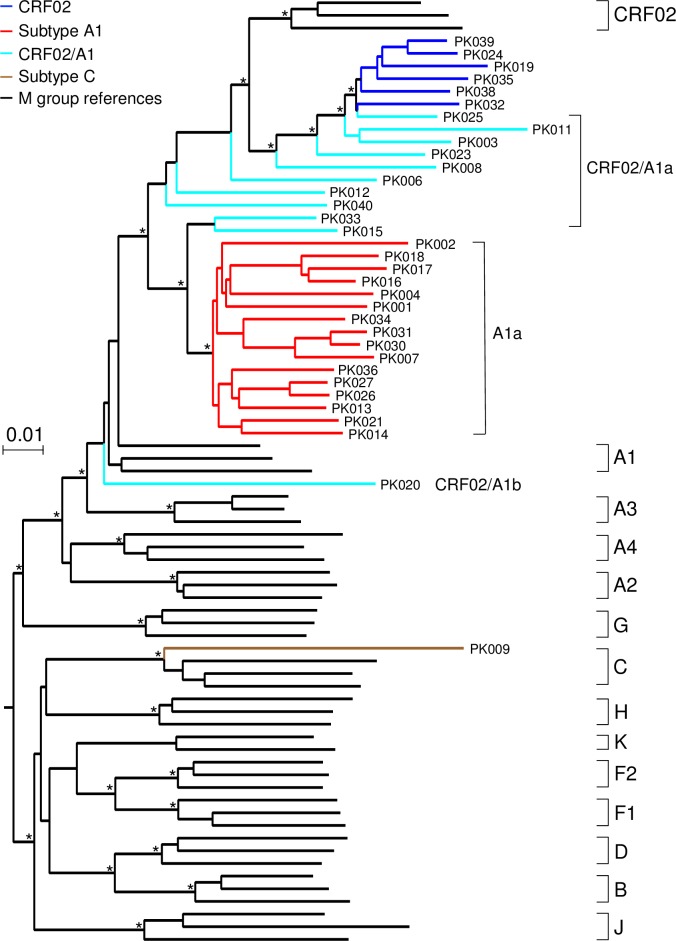
Phylogenetic tree analysis of near full-length genome sequences. Newly obtained NFLG sequences from 34 HIV-1 infected individuals from Karachi in Pakistan were aligned with subtype reference sequences from HIV-1 Sequence Database (www.hiv.lanl.gov). The phylogenetic tree was constructed using the Neighbor-Joining method and the Kimura two-parameter model. The scale bar represents 0.01 nucleotide substitutions per site. Asterisks indicate bootstrap values in which the cluster to the right is supported in 80% or more replicates (out of 1000). The subtype A1a, subtype C, CRF02 and CRF02/A1 recombinants are shown in red, brown, blue and cyan, respectively. Other subtype reference sequences are shown in black.

NFLG sequences were obtained from 28 plasma samples by amplifying two overlapping half genomes. Six samples that were negative for PCR amplification were co-cultured with PBMC to obtain virus isolates from which NFLG sequences were obtained from viruses in cell culture supernatants by PCR amplification of two overlapping half genomes or by the TruSeq RNA method (Table 1). NFLG amplification and virus isolation were not successful for the rest six samples. Phylogenetic analysis of 34 near full-length genome sequences together with subtype reference sequences showed that one was subtype C while all the others were related to subtype A1 and CRF02 (Fig 1). Similarity plot and bootscan analyses showed that seven NFLG sequences shared similar recombinant breakpoints as those in CRF02 (S1 Fig). However, eight other NFLG sequences only share some of the recombinant breakpoints in CRF02 (S1 Fig), suggesting that they were recombinants between subtypes A1 and G.

Phylogenetic analysis of 16 NFLG sequences (47%) formed a tight cluster within subtype A1 clade (Fig 1). This suggested that they were derived from one common subtype A1 ancestor in Pakistan and were named as subtype A1a. To investigate whether sequences obtained from previous studies also clustered with subtype A1a and were the result of the same introduction, we obtained all available HIV-1 sequences reported from Pakistan in the GenBank and compared them with the newly characterized subtype A1a sequences from this study. Since previous reported sequences were mainly generated for the partial *gag*, *pol* or *env* gene, we constructed three independent phylogenetic trees to study the relationship among all sequences in Pakistan. Phylogenetic tree analysis showed that all 16 A1a sequences from this study and nearly all subtype A1 sequences from other studies formed a tight subcluster in all three gene regions within the subtype A1 cluster (S2 Fig). These results showed that all these A1a viruses in Pakistan were derived from a single introduction.

The sequence from subject PK020 did not cluster with A1a or any A1 reference sequences. Since PK020 was as divergent as any other subtype A1 sequences (Fig 1 and S2 Fig), it was named as A1b. Examination of partial sequences from other studies also identified a few additional highly divergent subtype A1 variants (S2 Fig). Subtype assignment determined by the neighbor-joining method was confirmed by the Maximum Likelihood and Bayesian methods. These results indicated that other than the predominant A1a viruses, there were a number of other introductions of subtype A1 viruses into Pakistan. However, those viruses did not result in further dissemination as A1a. Instead, they might represent dead-end introductions.

### CRF02/A1 recombinants predominated the parental CRF02 viruses

**Fig 2:**
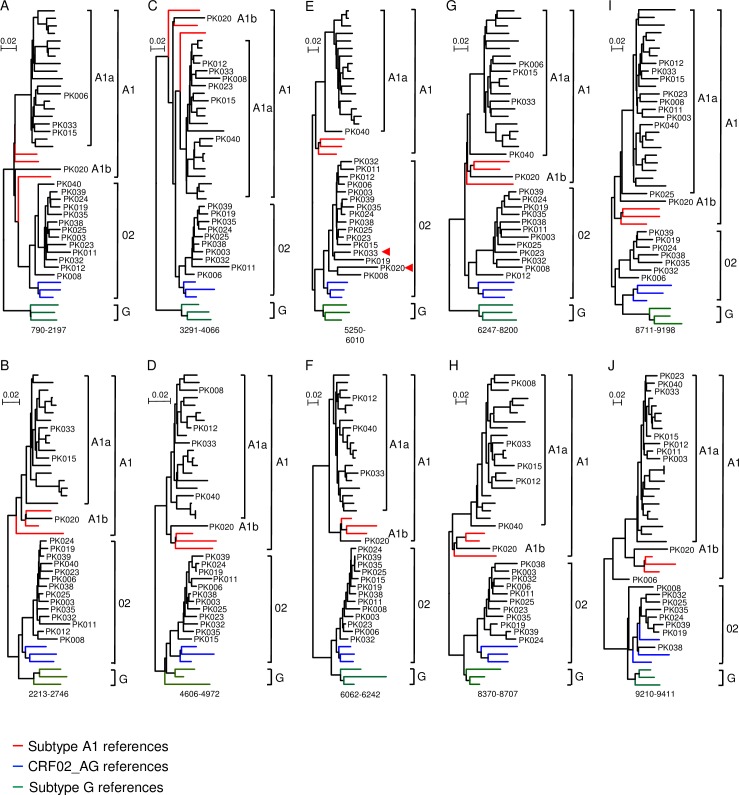
Phylogenetic tree analysis of recombinant fragment sequences in newly characterized viral genomes. All 34 subtype A1 and CRF02/A1 recombinant NFLG sequences were aligned with representative subtype A1, subtype G and CRF02 sequences. Phylogenetic trees were constructed for each of 10 recombination fragments in the CRF02 genome using the Neighbor-Joining method and the Kimura two-parameter model. The size of each recombinant region based on the location in the HxB2 genome is indicated at the bottom of the tree. The scale bar represents 0.02 nucleotide substitutions per site. The subtype A1, CRF02 and subtype G reference sequences are shown in red, blue and green, respectively, while 16 A1a sequences are shown in black. All 17 CRF02/ A1 recombinants are indicated with their sequence IDs. The CRF02-like subtype A recombinant sequence in the *vif/vpr* region in PK020 and PK033 are indicated by red triangles.

The initial analysis showed that 15 NFLG sequences were recombinants between CRF02 and A1a (S1 Fig). To understand how those recombinants were generated, we next investigated origins of the recombinant regions in their genomes. Since recombination occurred only between subtypes A1 and G as in the CRF02 genomes, three representative sequences for A1, G or CRF02 were analyzed together with all newly obtained NFLG sequences, except the subtype C sequence PK009. Interestingly, recombination analysis showed that the majority of recombination breakpoints in newly characterized recombinant sequences were similar to those in CRF02. To more clearly define the origins of these recombinant regions, we constructed phylogenetic trees for the minimum length sequences that were shared among recombinants at all 10 recombinant regions (A-J) (Fig 2).

**Fig 3:**
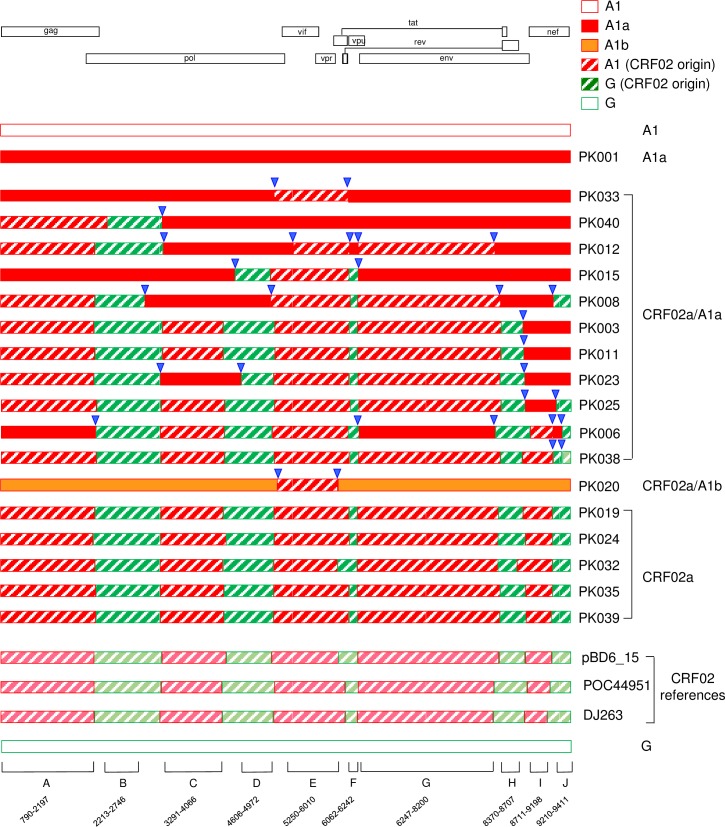
Recombination patterns of newly identified sequences. Recombination breakpoints were determined based on the analysis results with similarity plot, jpHMM and BootScan and recombination patterns for each NFLG genome was mapped using RecDraw. Subtype A1 and G references are indicated as red and green open box at the top and the bottom, respectively. One subtype A1a reference is shown in closed red bar. Three CRF02 (light color) and five CRF02a (dark color) are indicated as hatched bar with subtype A regions in red and subtype G regions in green. The subtype A1b sequence is shown in orange. The sizes of recombinant regions used for phylogenetic analysis are indicated based on the positions in the HxB2 genome. Recombination breakpoints between CRF02 and subtype A1 are indicated with blue triangles.

Recombinant fragment sequences from 16 A1a viruses (PK001, PK002, PK004, PK007, PK013, PK014, PK016, PK017, PK018, PK021, PK026, PK027, PK030, PK031, PK034 and PK036) always clustered together within the A1 cluster at all 10 regions (Fig 2). This further confirmed that A1a sequences share the same ancestor. Similarly, all 10 recombinant region sequences from six CRF02 viruses also formed a tighter subcluster (CRF02a) which was more closely related to the CFR02 sequences than to subtype G sequences, even in the regions derived from subtype G (Fig 2B, 2D, 2F, 2H and 2J). Analysis of all available partial *gag* and *pol* sequences of CRF02 viruses from Pakistan in the database together with the newly characterized NFLG sequences from this study also showed a tight subcluster of all CRF02 sequences with Pakistan origin (S3 Fig). These results demonstrated that both subtype A1a and CRF02a had evolved into unique sequences specific for Pakistan after they were introduced into the country as subtype B’ sequences in Thailand [26]. Examination of sequences in the same 10 recombinant region sequences in 10 other CRF02/A1 recombinants showed that the subtype A regions clustered with either A1a or CRF02a-like A regions in CRF02 (Fig 2A, 2C, 2E, 2G and 2I) while the CRF02 regions always formed a tight cluster together with CRF02a sequence that were identified only in Pakistan (Fig 2B, 2D, 2F, 2H and 2J), except that PK006 and PK038 branched out from the Pakistan specific subcluster in subtype G region in the last part of the *nef* gene (Fig 2J). More detailed analysis of the 3’-half sequences of the *nef* gene showed that PK006 was a recombinant between A1a and CRF02a in the 3’-half of the *nef* gene while PK038 represented a recombinant between CRF02a and prototype CRF02 (Fig 3 and S4 Fig). However, no sequences from any of these regions clustered with subtype A1 or subtype G.

The phylogenetic tree and recombination analyses of NFLG sequences showed that PK020 represented a divergent A1b sequence (Fig 1 and S2 Fig). Exploratory phylogenetic tree analysis of 10 recombinant region sequences confirmed that sequences from nine regions did not cluster with any reference sequences or newly characterized sequences. However, it clustered tightly together with CRF02a sequences in the third CRF02-like subtype A region (*vif/vpr)* (Figs 2E and 3). Similar analysis showed that sequences from nine of these regions in PK033 clustered with A1a sequences. However, like PK020, it also clustered tightly together with CRF02a sequences in the same third CRF02-like subtype A region (*vif/vpr)* (Figs 2E and 3). These results demonstrated that both PK020 and PK033 were recombinants; while the most parts of PK020 and PK033 genomes were A1b and A1a, respectively, both recombined with CRF02a that had evolved into unique Pakistan-specific virus population at middle of the viral genome (*vif/vpr)*.

Taken together, analysis of 17 CRF02/A1 recombinant NFLG sequences showed that five were CRF02a that had evolved into a subpopulations of sequences unique for Pakistan viruses, while 12 others were recombinants that were generated between CRF02a and A1a or A1b sequences that were only circulating in Pakistan. However, recombination patters in these 12 viruses were different from each other, except in PK003 and PK011 (Fig 3). These results suggested that newly generated CRF02/A1 recombinants had overtaken the prototype CRF02 viruses in this cohort.

### Timing of introductions of A1a and CRF02a into Pakistan

**Fig 4:**
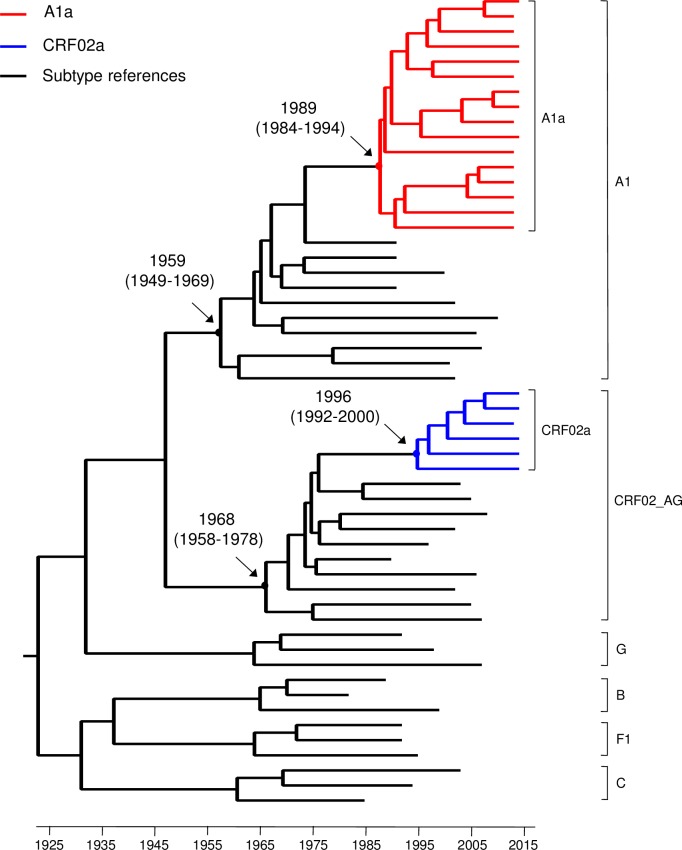
Estimated time of introduction of subtype A1a and CRF02 into Pakistan. A total of 57 near full-length genome sequences were used for the analysis. Among them, 16 A1a (red) and 6 CRF02a (blue) were newly characterized HIV-1 sequences in this study, while 35 were references sequences with known sample dates from Los Alamos HIV Sequence Database. Maximum-clade credibility trees were generated using the Bayesian MCMC approach implemented in BEAST1.8.2. Each Markov Chain Monte Carlo (MCMC) analysis was run for 50 million steps and sampled every 10,000 states. Posterior probabilities were calculated with a 10% burn-in and checked for convergence using Tracer v1.6. FigTree 1.4.2 was used for visualization of the annotated trees. The mean time and 95% highest posterior density (HPD) of the most common ancestor (tMRCA: year) were showed for the key notes based on relaxed (uncorrelated lognormal) molecular clocks under HKY nucleotide substitution models in a gamma-distribution of among site rate heterogeneity with four rate categories (HKY+γ4). All posterior probability values for key nodes are 1.0.

To estimate the timing of introduction of A1a and CRF02a viruses in Pakistan, we generated the maximum clade credibility (MCC) tree with NFLG sequences of 16 A1a sequences, 6 CRF02a sequences and 35 M group reference sequences (A1, CRF02, G, B, F1 and C) using BEAST v1.8.2 as previously described [27–30]. Analysis of the sequences by TempEst demonstrated that they had a positive correlation between genetic divergence and sampling time (*R* ^2^ = 0.39), and thus were suitable for phylogenetic molecular clock analysis implemented in BEAST (S5 Fig). Estimations using the relaxed and strict molecular clocks with the HKY or GTR substitution model showed the similar results to the tMRCA of A1a and CRF02a in Pakistan (S1 Table). Phylogenetic reconstruction under the relaxed clock with the HKY substitution model showed that the time to the most recent common ancestor (tMRCA) for A1a viruses was 1989 [95% Highest posterior density (HPD): 1984–1994], and CRF02a viruses were introduced into Pakistan at a later time point, at 1996 (95% HPD: 1992–2000) (Fig 4). Both A1a and CRF02a sequences from Pakistan formed unique independent subclusters within subtype A1 and CRF02 clades, respectively. This further confirmed that Both A1a and CRF02a viruses evolved into unique subpopulation sequences specific for Pakistan after their introductions in late 80’s or mid 90’s.

### CRF02/A1 recombinants occurred after introductions of parental viruses into Pakistan

Although the recombination patterns were variable among 12 NFLG recombinant sequences, only four genotypes (A1a, A1b, CRF02a and CRF02) were involved in recombination; 10 between A1a and CRF02a (PK003, PK006, PK008, PK011, PK012, PK015, PK023, PK025, PK033 and PK040), one between CRF02 and CRF02a (PK038), and one between A1b and CRF02a (PK020) (Fig 3). Examination of origins of all recombination fragments showed that all but two (PK020 and PK038) were derived from the A1a and CRF02a sequences that formed an unique virus population specific for Pakistan after they were introduced into Pakistan. Even in the two exceptions (PK020 and PK038), CRF02a was one of the recombination partners (Fig 3). No recombinant fragment sequences were found derived from pure subtypes A1 and G. This demonstrated that all 12 CRF02/A1 recombinants were generated between subtype A1 and CRF02 after both were introduced into Pakistan and evolved into subpopulation sequences specific for Pakistan.

### Similar recombination breakpoints as in CRF02 viral genomes

Examination of all recombination breakpoints showed that the majority of them were similar to the positions in the prototype CRF02 genomes (Fig 3). The recombinant breakpoints were even preserved for a very small recombinant region in the *vpu* gene in PK012, which contained five recombinant breakpoints in the genome (Fig 3 and Fig 2F). PK003 and PK011 shared the same recombination breakpoint between CRF02a and A1a in the genome (Fig 3). However, phylogenetic tree analysis showed that sequences in all recombination regions were never closer to each others than to other sequences in the subclusters (Fig 2). These showed that although PK003 and PK011 share the same recombination pattern, both were not derived from each other. Instead, they represented independent recombination events. These unique recombination genome patterns among the majority of the new CRF02/A1 recombinants indicated ongoing recombination among the circulating viruses in Pakistan.

## Discussion

**Fig 5:**
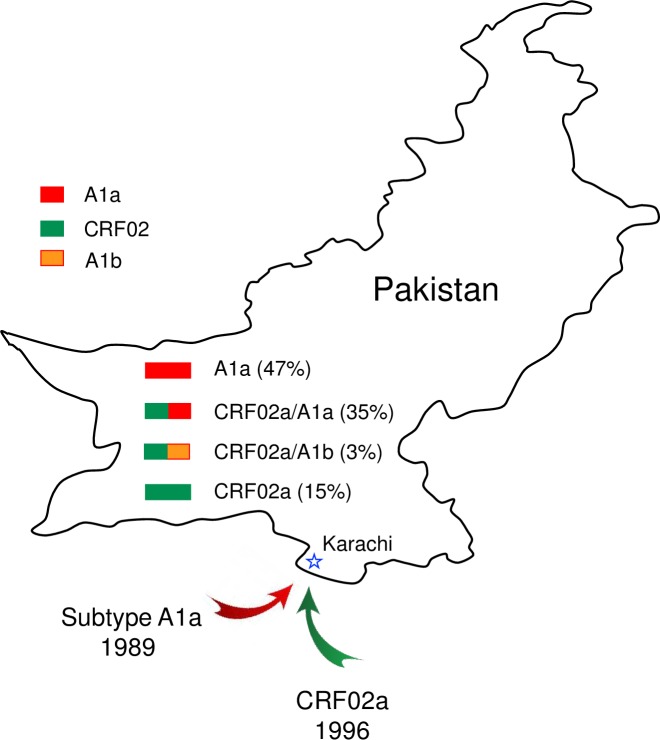
Distribution of different genotypes of newly characterized viruses in Karachi, Pakistan. The introduction time of subtype A1a and CRF02a are indicated. Sequences derived from subtype A1a, subtype A1b and CRF02a are shown in red, orang and green, respectively.

Analysis of NFLG sequences from 34 HIV-1-infected individuals in Karachi, Pakistan showed a high rate (38%) of new recombinant viruses (Fig 5). This is significantly different from what was reported in literature and the HIV-1 sequence database [9–11]. Analysis of all available partial sequences in the database showed that only 2.6% of viral sequences were A1/G recombinant (excluding CRF02 sequences). The much higher rate of recombinant viruses in this study suggests the recombination is actively generated among co-circulating viruses and have overtaken one prototype CRF02 virus and reduced the percentage of subtype A1 viruses (47% vs. 84%) at least in this cohort. NFLG sequences in other cities are needed to confirm if such a high rate of recombinant viruses exists at the national level. One reason for detecting much higher percentages of recombinant viruses in Pakistan is that analysis of NFLG sequences is much more sensitive and accurate for detection of recombinant HIV-1 genomes [31].

Molecular evolution molecular clock analysis of NFLG sequences showed that subtype A1 and CRF02 viruses were introduced into Pakistan in 1989 and 1996, respectively. The tight clusters of subtype A1 or CRF02 sequences, which could easily be distinguished from prototype subtype A1 and CRF02 sequences from other countries, suggested that they were results of single introductions and both evolved into unique virus populations specific for Pakistan after their introductions. This result is in agreement with the previous study that showed a “founder effect” of subtype A1 sequences in Pakistan [32]. The detection of a divergent A1b sequence (PK020) in this study and a few similar divergent subtype A1 sequences indicates introductions of other subtype A1 viruses. However, those viruses did not disseminate and likely became dead-end introductions. Analysis of the origins of each recombinant fragments showed that the vast majority of them were from A1a or CRF02a viruses, and none of them were derived from pure subtype G or other A sub-subtypes. These results demonstrated that all those new recombinants were generated after the viruses had evolved into distinct viral populations in Pakistan.

Interestingly, nearly all recombination breakpoints in the new CRF02/A1 recombinants were similar to those in CRF02, indicating recombination at these sites might likely generate recombinant viruses that were viable or had better replication advantage than parental viruses. A previous study has shown that CRF02 had a higher replicative capacity than its parental subtypes A and G *in vitro* [33]. New CRF02/A1 NFLG recombinant sequences (12) were found to be two times more than the parental CRF02a viruses (5). Moreover, the most of the recombinant genome patterns in these new CRF02a/A1a recombinant genomes were different. These results suggest that a high level of recombination is ongoing among co-circulating viruses and those newly generated recombinants may become predominant strains in Pakistan.

Our results confirm that it is critical to analyze whole genome sequences to fully understand the distribution of different genotypes in any regions, especially in areas where multiple genotypes are co-circulating. Recent advances in improvement of reverse transcriptases, PCR amplification methods and high-throughput sequencing technology will make it possible to analyze whole HIV-1 genome sequences for more accurate molecular epidemiological surveys. The whole genome sequence analysis will be critical for a better understanding of HIV-1 distribution, origin, transmission and molecular epidemic patterns, as well as for preparedness of vaccine evaluation sites.

## Supporting Information

S1 Fig Recombinant genome patterns of newly characterized sequences. (PDF)

S2 Fig Phylogenetic tree analysis of all available subtype A1 sequences from Pakistan. (PDF)

S3 Fig Phylogenetic tree analysis of all available CRF02 sequences from Pakistan. (PDF)

S4 Fig Phylogenetic tree analysis of recombinant regions in the 3’-half *nef* gene. (PDF)

S5 Fig Root-to-tip regression to estimate the tMRCAs and clock rates. (PDF)

S1 Table Time to the most recent common ancestor of characteristics of near full-length genome sequences in Karachi, Pakistan. (PDF)
