# Supplementary material for: Fast Dissemination of New HIV-1 CRF02/A1 Recombinants in Pakistan
Source: PLoS One. 2016 Dec 14;11(12):e0167839. doi: 10.1371/journal.pone.0167839 (PMC5156399; doi:10.1371/journal.pone.0167839)
Supplement: S1 Fig — (PDF) [file pone.0167839.s001.pdf]

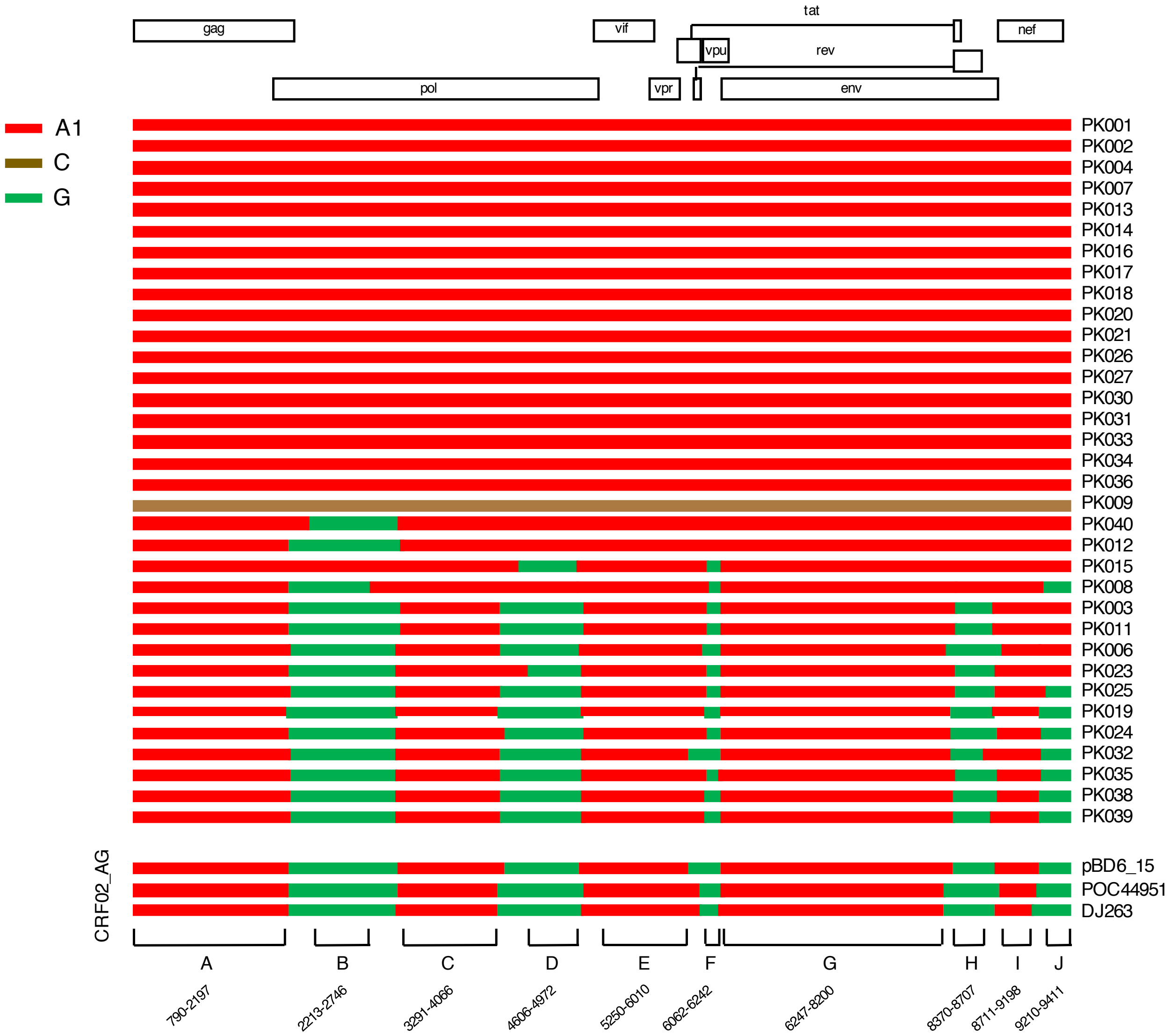

S1 Fig. Recombinant genome patterns of newly characterized sequences. Sequences with origins of subtype A1, subtype C and subtype G are shown in red, orang and green, respectively. Recombination patterns of three CRF02 references (pBD6_15, POC44951 and DJ263.10) were shown at the bottom. Ten subtype A and G regions (A through J) based on CRF02 genomes were used to determine origins of sequences in each region. The sizes of recombinant regions used for phylogenetic analysis are indicated at the bottom based on the positions in the HxB2 genome.
