# Supplementary material for: Fast Dissemination of New HIV-1 CRF02/A1 Recombinants in Pakistan
Source: PLoS One. 2016 Dec 14;11(12):e0167839. doi: 10.1371/journal.pone.0167839 (PMC5156399; doi:10.1371/journal.pone.0167839)
Supplement: S2 Fig — (PDF) [file pone.0167839.s002.pdf]

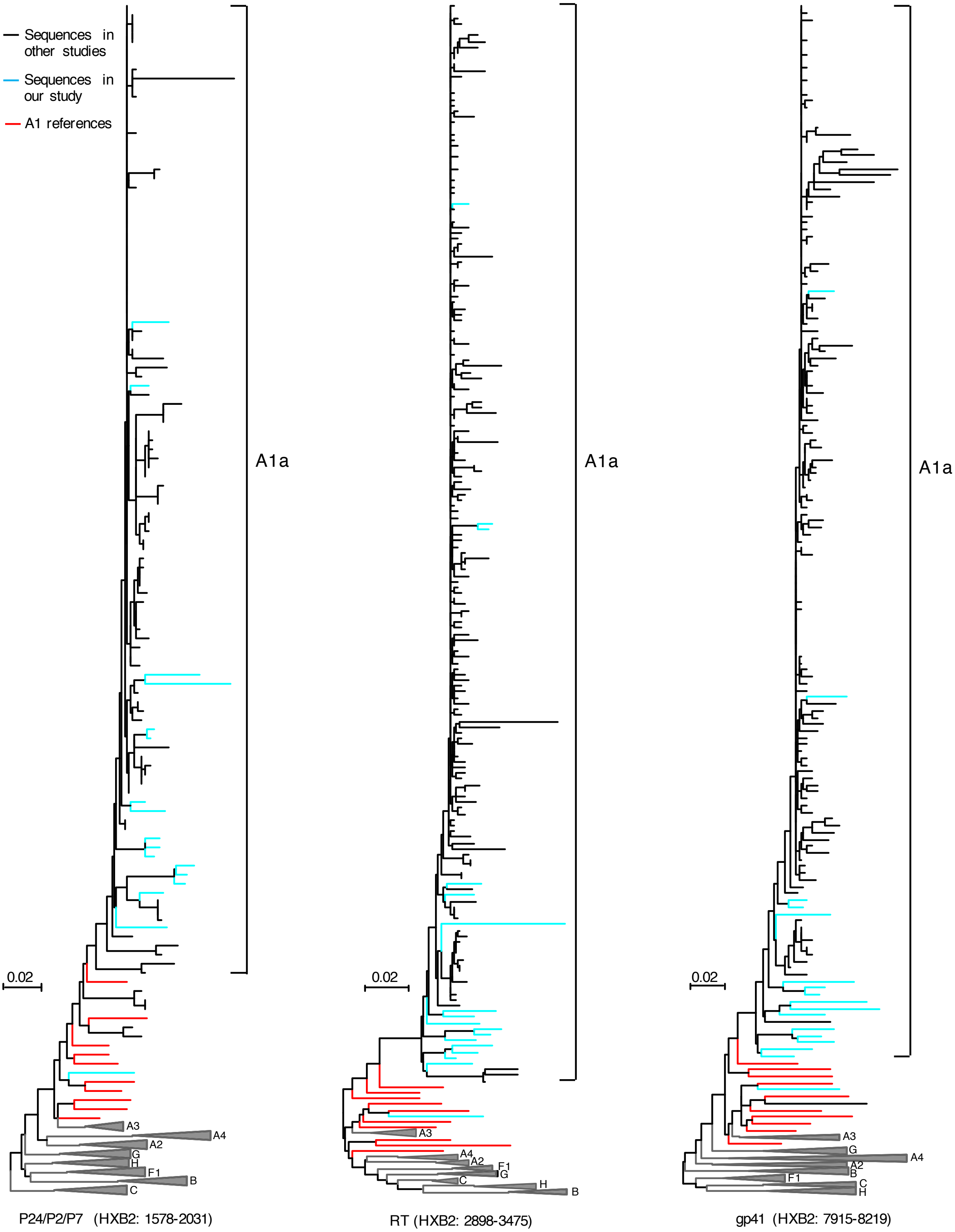

S2 Fig. Phylogenetic tree analysis of all available subtype A1 sequences from Pakistan. Partial gag (A), pol (B) and env (C) sequences (>300bp) available from Pakistan in the HIV sequence database were analyzed together with the newly characterized subtype A1 sequences from this study. Since these sequences did not overlap each other, three Neighbor-Joining trees were constructed to include as many sequences as possible and to maximally utilize the sequence length for all available sequences. The newly characterized subtype A1 sequences from this and other studies are indicated in cyan and black, respectively. Subtype A1 references sequences are shown in red. Other subtype references sequences are shown as collapsed subtrees.
