# Supplementary material for: Fast Dissemination of New HIV-1 CRF02/A1 Recombinants in Pakistan
Source: PLoS One. 2016 Dec 14;11(12):e0167839. doi: 10.1371/journal.pone.0167839 (PMC5156399; doi:10.1371/journal.pone.0167839)
Supplement: S3 Fig — (PDF) [file pone.0167839.s003.pdf]

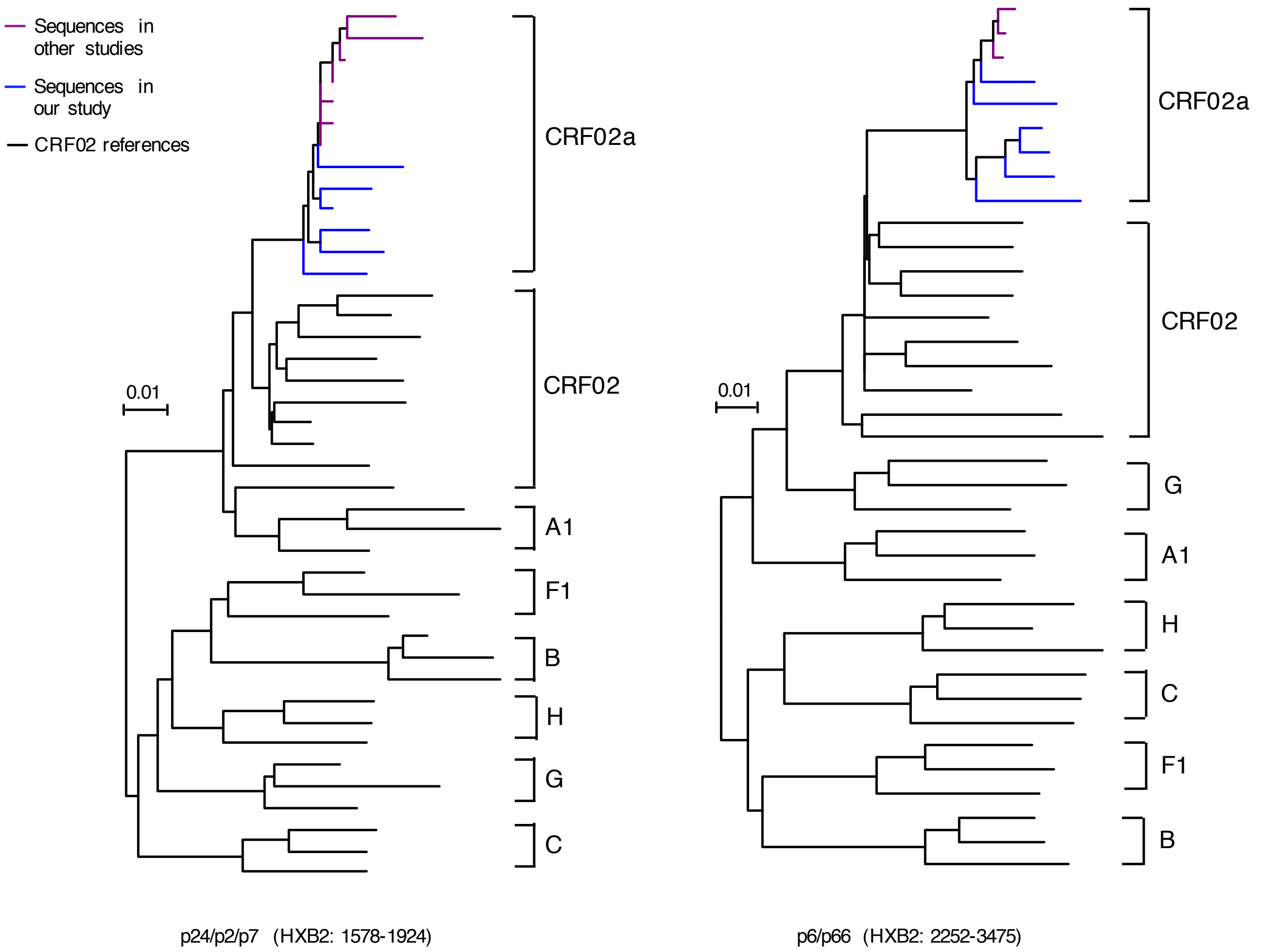

S3 Fig. Phylogenetic tree analysis of all available CRF02 sequences from Pakistan. Partial *gag* (A) and *pol* (B) sequences (>300bp) available from Pakistan in the HIV Sequence Database were analyzed together with the newly characterized CRF02 sequences from this study. The newly characterized CRF02 sequences from this study and other studies are indicated in blue and purple, respectively. Subtype reference sequences are shown in black.
