# Supplementary material for: Fast Dissemination of New HIV-1 CRF02/A1 Recombinants in Pakistan
Source: PLoS One. 2016 Dec 14;11(12):e0167839. doi: 10.1371/journal.pone.0167839 (PMC5156399; doi:10.1371/journal.pone.0167839)
Supplement: S4 Fig — (PDF) [file pone.0167839.s004.pdf]

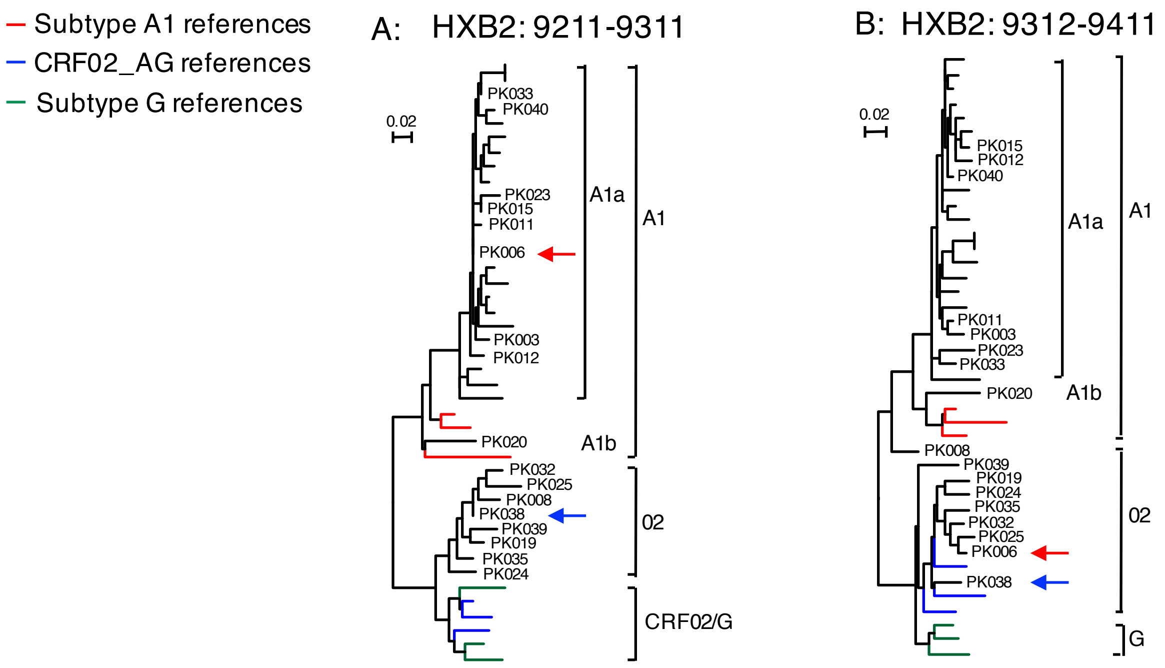

S4 Fig. Phylogenetic tree analysis of recombinant regions in the 3'-half nef gene. The phylogenetic tree was constructed with the two sequence fragments in 3'-half nef sequences to show recombination between subtype A1a (A) and CRF02a (B) in PK006 (indicated by red arrow) as well as between CRF02a (A) and prototype CRF02 (B) in PK038 (indicated by blue arrow). The recombinant PK006 sequences are indicated by red arrow. Subtype A1, CRF02 and subtype G reference sequences are shown in red, blue and green, respectively.
