# Supplementary material for: Fast Dissemination of New HIV-1 CRF02/A1 Recombinants in Pakistan
Source: PLoS One. 2016 Dec 14;11(12):e0167839. doi: 10.1371/journal.pone.0167839 (PMC5156399; doi:10.1371/journal.pone.0167839)
Supplement: S5 Fig — (PDF) [file pone.0167839.s005.pdf]

A
B

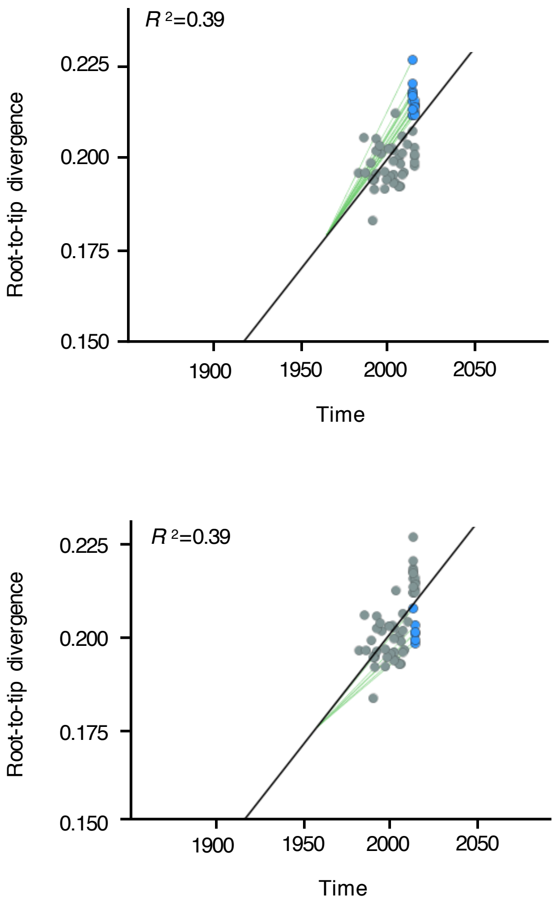


**S5 Fig. Root-to-tip regression to estimate the tMRCAs and clock rates.** A simple linear regression of the root-to-tip genetic distances against the sampling dates was performed using the TempEst software. The root was determined by maximizing the coefficent of determinant R2. The vertical axis measures the genetic distances between the samples and the root while the horizontal axis scales the sampling time (year). The same sequence alignment of the newly characterized 22 NFLG sequences from Pakistan and 35 subtype reference sequences from Los Alamos HIV Sequence Database (www.hiv.lanl.gov) was analyzed. Ancestor traces were shown in green lines for subtype A1a (the blue dots in panel A) and CRF02a (the blue dots in panel B).
