# Supplementary material for: Fast Dissemination of New HIV-1 CRF02/A1 Recombinants in Pakistan
Source: PLoS One. 2016 Dec 14;11(12):e0167839. doi: 10.1371/journal.pone.0167839 (PMC5156399; doi:10.1371/journal.pone.0167839)
Supplement: S1 Table — (PDF) [file pone.0167839.s006.pdf]

S1 Table. Time to the most recent common ancestor of characteristics of near full-length genome sequences in Karachi, Pakistan

Genotype	HKY+Γ_4 constant size		GTR+Γ_4 constant size	
	Relaxed Model	Strict Model	Relaxed Model	Strict Model
Subtype A1	1989 (1984-1994)	1983 (1979-1986)	1990 (1985-1995)	1983(1980-1986)
CRF02_AG	1996 (1992-2000)	1994 (1992-1996)	1997 (1993-2001)	1994 (1992-1996)

Time to the most recent common ancestor (tMRCA: year), the 95% highest posterior density (HPD) confidence intervals are given in parentheses.
